# Transcriptomic Data Meta-Analysis Sheds Light on High Light Response in *Arabidopsis thaliana* L.

**DOI:** 10.3390/ijms23084455

**Published:** 2022-04-18

**Authors:** Aleksandr V. Bobrovskikh, Ulyana S. Zubairova, Eugeniya I. Bondar, Viktoriya V. Lavrekha, Alexey V. Doroshkov

**Affiliations:** 1 Institute of Cytology and Genetics Siberian Branch, Russian Academy of Sciences, 630090 Novosibirsk, Russia; avb@bionet.nsc.ru (A.V.B.); ulyanochka@bionet.nsc.ru (U.S.Z.); vvl@bionet.nsc.ru (V.V.L.); 2 Institute of Computational Mathematics and Mathematical Geophysics Siberian Branch, Russian Academy of Sciences, 630090 Novosibirsk, Russia; 3 Institute of Fundamental Biology and Biotechnology, Siberian Federal University, 660036 Krasnoyarsk, Russia; ebondar@sfu-kras.ru

**Keywords:** high light stress, transcriptomic data, *Arabidopsis thaliana* L., gene network, transcription factors, transcription factors binding sites, systems biology, meta-analysis

## Abstract

The availability and intensity of sunlight are among the major factors of growth, development and metabolism in plants. However, excessive illumination disrupts the electronic balance of photosystems and leads to the accumulation of reactive oxygen species in chloroplasts, further mediating several regulatory mechanisms at the subcellular, genetic, and molecular levels. We carried out a comprehensive bioinformatic analysis that aimed to identify genetic systems and candidate transcription factors involved in the response to high light stress in *Arabidopsis thaliana* L. using resources GEO NCBI, string-db, ShinyGO, STREME, and Tomtom, as well as programs metaRE, CisCross, and Cytoscape. Through the meta-analysis of five transcriptomic experiments, we selected a set of 1151 differentially expressed genes, including 453 genes that compose the gene network. Ten significantly enriched regulatory motifs for TFs families ZF-HD, HB, C2H2, NAC, BZR, and ARID were found in the promoter regions of differentially expressed genes. In addition, we predicted families of transcription factors associated with the duration of exposure (RAV, HSF), intensity of high light treatment (MYB, REM), and the direction of gene expression change (HSF, S1Fa-like). We predicted genetic components systems involved in a high light response and their expression changes, potential transcriptional regulators, and associated processes.

## 1. Introduction

Sunlight is one of the key factors for plant growth and development. Understanding how plants can deal with different lightning regimes using their adaptive capabilities on the molecular genetic and metabolic levels is a crucial fundamental task. At the same time, knowledge about possible regulators of these adaptations could help in the design of new plant varieties with an increased productivity in non-optimal light conditions. In particular, plants, being sessile organisms, have developed resistance to changing lighting conditions [1], but their adaptations have certain limits that depend on their evolution in a particular ecological niche. Thus, light conditions beyond these limits lead to stress and a decrease in plant productivity. Current knowledge related to the high light stress response of plants is incomplete and the systems’ biology approach could help in order to produce a detailed description of the molecular genetic systems involved in such processes and to find new candidate transcription factors (TFs) more precisely.

The sensing of high light is complex and includes the plant response at different levels. The review [2] considered the main strategies for sensing and responding to excess light. Authors emphasized various types of sensing mechanisms, including photoreceptors regulations, chloroplast avoidance movements, changes in the redox state in the cell (e.g., pH of thylakoid lumen), accumulation of metabolites, non-photochemical quenching, and systemic gained acclimation.

Photoreceptors have a special role in light-dependent regulations since they are involved in dowstream protein–protein interactions. The photoreceptor families are cryptochromes, phototropins, phytochromes, Zeitlupe, and UV-B resistance 8 (UVR8). Cryptochromes (CRY1/CRY2) can suppress the activity of the COP1 complex in response to blue light [3]. In addition, the interaction of CRY2 with SPA1 in blue light conditions mediates the suppression of the SPA1 complex. Other transmitters of blue light signals are phototropins (PHOT1, PHOT2 in *Arabidopsis thaliana* L.), which can relocalize in response to blue light from plasma membrane to the cytoplasm and Golgi apparatus, respectively [4]. Phytochromes and their interacting factors can inhibit the E3 ligase complex to stabilize the transcription factors (TFs) of light-growth development [5]. It was established that PIF3 (phytochrome interacting factor) triggers a reduction in phytochrome B, which leads to the degradation of phyB and PIF3 itself [6], which is the central step of phyB regulation. The ZEITLUPE family of proteins (ZTLs, FKF1, LKP2) is involved in the process of floral transitions and forms the E3 ligase complex [7], and seems to control light-dependent protein degradation [8,9,10]. Among photoreceptors, UVR8 has a special role in UV-B-dependent acclimation; it was shown that UVR8 homodimers monomerized upon UV-B absorption and interacts with COP1 (which regulates positively through the HY5 factor), resulting in the activation of photomorphogenetic processes and the accumulation of flavonoids [11]. This process can be repressed by WD-40 proteins (RUP1 and RUP2), which cause the redimerization of UVR8. Recent work [12] revealed that cryptochromes CRY1 and CRYB could induce RUP1 and RUP2, which depends on HY5.

Thus, the main downstream targets of photoreceptors are HY5, E3 ligase, COP1 and SPA1 complexes and RUP1/RUP2 proteins, which cause further changes in the expression of their target genes. For instance, transcription factor HY5 of the bZIP family activates photomorphogenic development by directly binding with promoters of light-inducible genes [13]. It is well known that the E3 ligase complex of COP1 and SPA can suppress photomorphogenesis in dark conditions and regulate photoperiodic flowering [14]. COP1 interacts with the floral regulator CO and causes CO ubiquitination and degradation [15]. In addition, we know some members of subcellular signal transduction pathways in response to high light, such as GUN1, STN7, EX1/EX2 (chloroplasts), PHOT2 (cytosolic), CRY1, and ABI4 (nuclear) [2]. Some target genes of these inductors (*ABI4*, *ZAT10*, *ZAT12*, *LHCB*, *EX1*, *EX2*, and *CRY2*) are well-known to induce antioxidant genes (*APX1*, *APX2*) and WRKY and ELIP factors. The study of [1] discusses the importance of chromatin modification in the process of the response to light, which leads to a change in the expression levels of the associated gene cascade, such as *CAB*, *RBCS1A*, and *GUN5*. Thereby, we know the key members of light-related regulation and some members of downstream pathways. However, we still lack comprehensive knowledge about its genetic regulation.

The primary damage caused by overlighting is related to the exponential production of reactive oxygen species (ROS). ROS are produced by many cellular organoids, including chloroplasts, mitochondria, peroxisomes, and even apoplast through the action of NADPH-oxidases [16,17,18,19]. Both non-photochemical and photochemical reactions produce active forms of oxygen under light stress. For instance, it has been shown that, during hyperinsolation, photosystem I produces a superoxide anion and, subsequently, peroxides and hydroxyl radical [20,21]. Further, this leads to the spreading of peroxides and oxidation of the whole cell, the oxidation of its lipid components, and the disruption of biochemical reactions [22,23,24]. To prevent non-specific cytotoxic damage to cells, plants have developed a system of enzymes and low-molecular-weight antioxidants that help to resist extreme oxidative stress [25,26]. On the other hand, ROS and cytoplasmic oxidation processes are components of the ROS-related signaling system involved in responding to abiotic stresses by the direct activation of antioxidant systems, as well as more complex responses [27,28].

Apart from genetic factors involved in the light response of plants, there are complex multilevel reactions in the plant response. It was noted that secondary metabolites, such as isoprenoids and phenylpropanoids, have an essential role in maintaining the antioxidant system in conditions of severe excess light stress [19]. The photoinhibition of photosystems I/II during high light stress is a huge bottleneck because of its damage [19]. The state of photosystem II protein D1 is crucial for plants surviving during excessive light treatment because of its degradation. Authors [29] pointed out the importance of DegP proteases and the FtsH proteases in restoring protein D1. Still, the question about the repair of the PSI complex remains open. Another type of reaction, whose mechanism is not fully understood, is the coordination of the stomatal response and leaf-to-leaf communication during light stress [30].

However, there are no standard protocols for the study of high light treatment: the duration and intensity of the treatment can vary from minutes to days and from 500 to over 2000 μmol m${}^{-2}$ s${}^{-1}$, respectively. There is also the correlation between light intensity and temperature, which can lead to an intersection between both types of stresses. Therefore, for a comprehensive study of light stress, we should take into consideration heterogeneous conditions of single experiments (treatment duration, intensity of light, and wavelength). In this way, the suitable way to study the relationship between genes involved in the light stress response is using transcriptomic data from different timepoints and various intensities of high light in conjunction with all available information on gene interactions. It was shown that the core gene network of the high light response included approximately 150 genes, considering various databases [31]. Such knowledge can be useful as a basis for further investigations of new components and their interactions. For example, a review of [32] described the details of the dynamic of ABA-regulatory network and summarized the knowledge about individual regulators and their roles. In addition, omics technologies could shed light on the role of particular pathways involved in various light conditions; for instance, the work of [33] provides detailed information about the regulation of CAM-related pathways in dark, normal, and hyperinsolation conditions. On the other hand, new knowledge about transcriptional regulators can be obtained by using a meta-analysis of transcriptional data followed by a search for promoter signals in corresponding networks [34].

The benefits of the integrative approach for revealing new genes involved in regulation are shown by [35]. In particular, the use of fluorescence-activated nucleus sorting and laser-capture microdissection with next-generation RNA sequencing helped to identify over 15,000 tissue-specific differentially expressed genes (DEGs) and reveal their TFs on a genome-wide scale. Therefore, the general usage of different data types for such types of work is a modern and suitable approach. On the other hand, transcriptomic data combined with multivariate data analysis and statistics is a powerful approach for describing regulation patterns of individual gene families. For instance, such an approach was useful for studying antioxidant gene dynamics in response to stresses (drought, cold) based on transcriptomic data and qPCR gene profiling [36]. As a result, authors identified prospective antioxidant genes involved in the stress response.

Therefore, the plant reaction to light stress is complex and involves many genes and their regulators, and their further study will expand fundamental aspects of related gene networks and will allow us to predict new interactions and implicit interconnections with other systems. Therefore, the integration of data on transcriptomic regulation and interactions of genes and their products is supposed to be a fruitful approach. Thus, the aim of this study is to reveal the response of the gene network of high light stress based on available data for model organism *Arabidopsis thaliana* L., including transcriptomic experiments for high light stress in combination with genetic network and multidimensional approaches for revealing trends of complex regulation.

## 2. Results

### 2.1. A Pipeline for Large-Scale Systematic Analysis of the High Light Response Regulation at Different Levels

**Figure 1:**
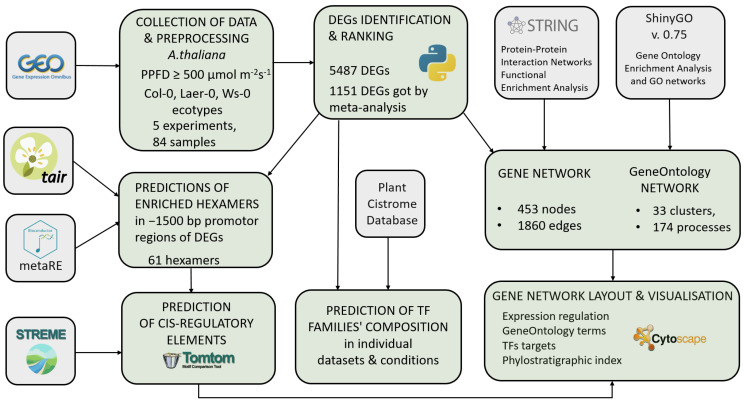
Interaction of data searching and processing steps in the framework large-scale systematic analysis of the high light response regulation at different levels.

For the present large-scale systematic analysis, the data were searched and processed with the following interacting stages (see general scheme in Figure 1): (i) meta-analysis of several transcriptomic experiments from NCBI GEO DataSets database (www.ncbi.nlm.nih.gov, date of access 5 January 2022) and predicting a set of most likely differentially expressed genes and their ranking based on the significance of the expression change in response to high light; (ii) prediction of transcription factor families in individual datasets using the Plant Cistrome Database [37]; (iii) prediction of enriched hexamers in a set of 1151 selected DEGs and identification of corresponding cis-regulatory elements using STREME [38] and Tomtom tools [39]; (iv) reconstruction of protein–protein network according to String database [40] and GeneOntology network [41] based on 1151 selected DEGs. The following sections present the details of the results obtained.

### 2.2. The High Light Response Differentially Expressed Genes

#### 2.2.1. Sets of DEGs Predicted from Individual Transcriptomic Datasets

The meta-analysis of five transcriptomic experiments from the NCBI GEO DataSets database (www.ncbi.nlm.nih.gov, date of access 5 January 2022), including 84 samples in 25 treatments with different intensities and durations of high light action (see Table 1), allowed us to identify 5487 DEGs with FDR < 0.05, wherein 3533 DEGs were unique (see Supplementary Table S2).

**Figure 2:**
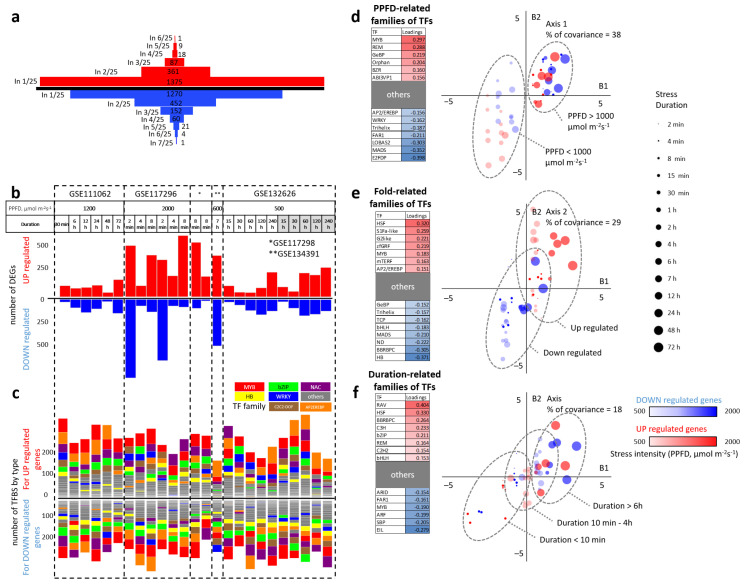
Sets of up- and downregulated differentially expressed genes (DEGs) allowed us to identify the correspondence between enriched transcription factor families and directions of expression changes, as well as PPFD levels and duration of high light expose in treatments. (**a**) The distribution of DEGs between datasets (up- and downregulated genes are marked by red and blue, respectively); (**b**) the distribution of upregulated DEGs (marked by red) and downregulated DEGs (marked by blue) in individual experimental datasets indicated by PPFD levels and duration of high light exposure (gray color indicate the Ws-0 ecotype); (**c**) the distribution of the enriched transcription factor families corresponding to individual experimental datasets; (**d**–**f**) the results of a multivariate analysis between experimental conditions (B1) and the sets of transcriptional regulators (B2) by the 2B-PLS method. The percentages of covariance for each axis, as well as families of transcription factors that have more than 0.15 of load modulus, are shown in the corresponding scatter plots. The point size represents the treatment duration. The point intensity represents the PPFD. Red color corresponds to upregulated genes, blue color corresponds to downregulated genes. The main differentiator of the first axis (**d**) is the light intensity. The second axis (**e**) distinguishes between upregulating and downregulating DEGs. The third axis (**f**) classifies the treatment by their duration.

The number of genes in different individual experiments and intersections of their sets are shown in Figure 2a,b. The largest amounts of downregulated genes were identified in the datasets related to the shortest (2 min) exposure time (Figure 2b).

#### 2.2.2. Sets of Enriched TFs Families for DEGs in Individual Datasets Considering the Direction of the Expression Change

The candidate TFs families among sets of individual DEGs have a heterogeneous composition between individual datasets, considering the direction of the expression change (Figure 2c). The most common TFs families are MYB, bZIP, NAC, HB, WRKY, C2C2-DOF, and AP2/EREBP, marking the stress response at the transcriptomic level. Such a heterogeneous composition and diversity of factors involved testifies in favor of the involvement of a large number of molecular genetic regulatory systems in response to stress, and also describes the differences between individual experimental conditions. Therefore, we conducted an additional analysis in order to identify trends in the composition of TFs (Figure 2d–f).

#### 2.2.3. Prediction of TFs Families Mediating the Direction of Expression Change and Response to Varying Intensity and Duration of High Light Exposure

The prediction of the relationship between experimental conditions and TFs families involved in regulating gene expression during the high light response was based on a two-block partial least squares method. As objects, we used the list of 50 individual experimental treatments (including varying the intensity and duration of high light exposure) resulting in changing of genes expression changing. The first block (B1) consisted of a quantified description of experimental conditions (the intensity, duration of light exposure, ecotype of samples, tissue, plant age, and direction of change in genes expression), and the second block (B2) included the number of significantly enriched TFs of each considered family (see Supplementary Table S4). The relationships between the individual peculiarities of datasets and the TFs composition is described in terms of covariance and shown in Figure 2d–f. The total amount of covariance described by the three revealed major factors is 85%, which speaks in favor of their strong impact toward the composition of TFs families. In particular, 38% of covariance is described by a factor of light intensity. Conditions of the highest light intensity (PPFD > 1000 μmol m${}^{-2}$ s${}^{-1}$) are strongly associated with an enrichment of MYB and REM TFs families, whereas the relatively low light intensity (PPFD between 500 and 1000 μmol m${}^{-2}$ s${}^{-1}$) is associated with E2FDP and MADS (Figure 2d). The second revealed factor is the direction of gene expression changes, which, in total, contributed to 29% of covariance. Upregulated genes are highly associated with HSF and S1Fa-like TFs families, whereas downregulated genes are associated with homeobox and BBRBPC TFs families (Figure 2e). The duration of high light treatment describes 18% of covariance. The short treatments (time < 10 min) are highly associated with EIL and SBP factors, whereas long treatments (time > 6 h) are associated with RAV and HSF factors.

#### 2.2.4. Set of the Most Significant DEGs Predicted by Meta-Analysis of Several Transcriptomic Experiments

We performed a meta-analysis of five studies of gene expression responses to high light stress in *Arabidopsis thaliana* L. (see Table 1) and revealed 5487 DEGs (FDR < 0.05), while a set of selected 1151 DEGs (see Supplementary Table S3) showed the greatest contribution to the overall pattern of expression changes. The annotation of these genes showed that many terms are significantly overrepresented, and there is a large number of specific categories related to high light (“response to light”, “response to UV-B”, “response to red light”, “response to ROS”, “response to JA”, “ethylene signaling”, “stress response”, “flavonoid biosynthesis”, “response to temperature”), which testifies the presence of a significant high light response component. A total of 33 metaterms clusters (see Supplementary Table S9) were identified by ShinyGO [41].

#### 2.2.5. Set of TFs Motifs Enriched in Promoter Regions of the Most Significant DEGs

**Figure 3:**
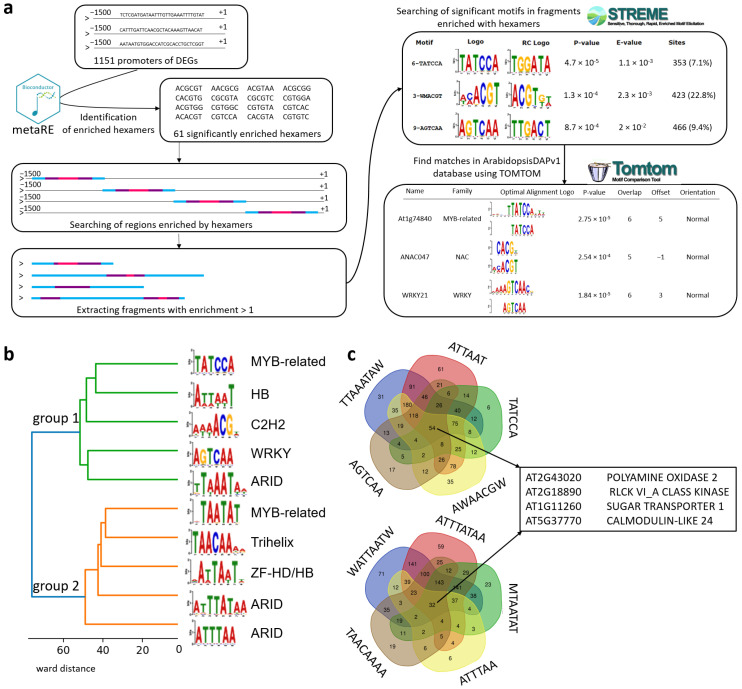
Prediction of enriched motifs in promoters of selected DEGs. Prediction of cis-regulatory elements in the upstream regions enriched with hexamers and their genetic targets based on *Arabidopsis thaliana* L. transcriptomic meta-analysis. (**a**) The general scheme of pipeline of transcriptome regulators predictions. Promoters 1500 bp upstream regions of top 1151 genes (from transcriptomic meta-analysis) were used to predict 61 overpresented hexamers (*p*-value < 0.05) (see Supplementary Tables S5–S7). After, 10 enriched motifs were identified by STREME and Tomtom tools (see Supplementary File S10). (**b**) Cluster analysis of identified motifs. (**c**) Venn diagrams for sets of genes for which isolated motifs are enriched in their promoter regions.

To evaluate the presence of TFs among all datasets, which helped us to predict the novel TFs related to general hyperinsolation, we separately performed an independent search in a set of DEGs with the greatest contribution (1151 genes). The overall steps are shown on (Figure 3a). In particular, 61 overrepresented hexamers were identified using the metaRE package. Then, the regions enriched with hexamers were extracted and scanned for known motifs using STREME. Revealed motifs were confirmed by the ArabidopsisDAPv1 database. Subsequently, 10 significantly overrepresented motifs belonging to the ARID, MYB, ZF-HD/HB, HB, Trihelix, WRKY, and C2H2 families were identified. They correspond to specific TF genes: *ATHB23*, *WOX11*, *AT5G66730*, *ANAC047*, *AT4G18890*, *AT3G13350*, *AT1G76110*, and *ANL2*. (Figure 3b). Three of them (MYB, WRKY, and HB TFs) were also significantly presented in individual experiments, which further validates our analysis of the TF composition.

According to their targets genes, the identified regulators are classified into two groups (clustering is shown in Figure 3b). The intersection of target genes is shown in Figure 3c on the right (Venn diagrams). Intersections within the groups testify in favor of a significant commonality of the targets of these motifs. On the other hand, for the identified central subsets of genes, only four genes are common in their composition between groups (their IDs and trivial names are also shown in Figure 3c), which is in favor of the specificity of targets in these subgroups. Therefore, the revealed enrichment in transcriptional regulators to hyperinsolation testifies in favor of the coordinated character of regulating this gene ensemble; therefore, the authors considered it promising to reconstruct the corresponding genetic network related to the response to high light intensity.

### 2.3. The High Light Response Core Gene Network

#### 2.3.1. General Structure of Functional Modules

**Figure 4:**
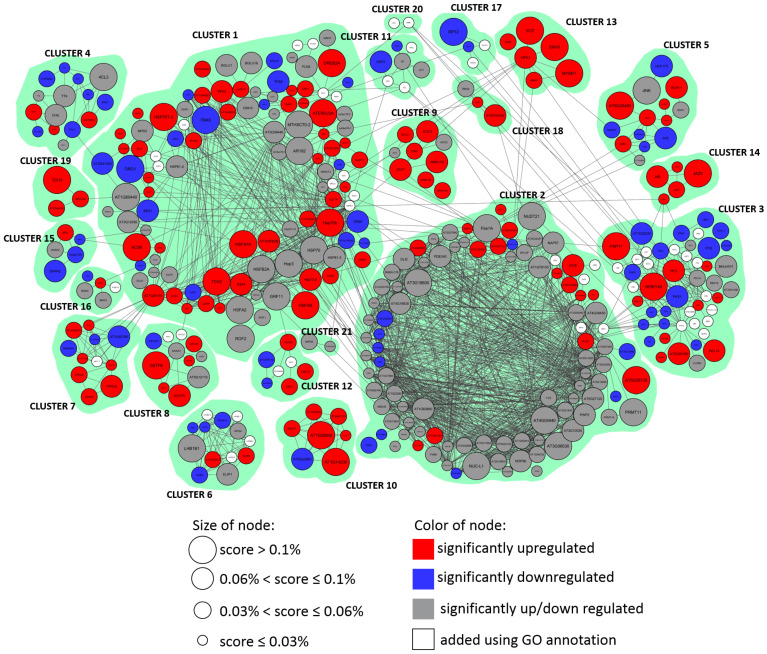
The reconstructed gene network split by 21 connectivity clusters corresponding to functional modules of high light stress response regulation.

The reconstructed gene network for the response to increased illumination in *Arabidopsis thaliana* L. comprises 453 nodes and 1860 edges, among which, the main part of the network includes 382 nodes and 1813 edges (see Figure 4). We divided this network into 21 clusters corresponding to functional modules (Table 2).

In general terms, the response to hyperinsolation presented as a gene network can be divided into specific and non-specific components. The non-specific component mainly includes clusters 1 and 2. The first cluster contains genes related to the side component of the response to high light stress, such as an increase in temperature: various heat shock genes, and genes with a response to reactive oxygen species; most of the genes included in it are characterized by an increase in expression. The second cluster contains mostly metabolic-related genes: 25 genes of ribosomal biogenesis, as well as genes responsible for the production and degradation of proteins. This cluster is also associated with a nonspecific response, but most of the nodes have multidirectional changes in expression, which shows its ability and adaptability. Other clusters that could describe the nonspecific response to hyperinsolation are according to the terms 5, 7, 8, 10, 12, 15, 16, and 18–21; most of them are associated with the first or second cluster and are located in the left part on the (Figure 3).

We have identified numerous clusters of a specific response to hyperinsolation, which are confirmed by their annotations. The specific response includes several components: the response to red/blue light, regulation of circadian rhythms, and jasmonic acid signaling. For instance, cluster 3 contains genes with a specific response to a light stimulus, including five genes of phytochromes and cryptochromes. In addition, two of the TFs identified by STREME and Tomtom are presented in this cluster. The fourth cluster is associated with metabolic processes and consists of flavonoid biosynthesis genes, phenylalanine biosynthesis genes, and phenylpropanoid synthesis pathway genes. The seventh cluster contains calcium-dependent protein kinases. The eighth cluster consists of antioxidant enzymes, in which, glutathione S-transferases are characterized by a strong tendency to upregulate.

Other clusters that could describe the specific response to hyperinsolation are according to the terms 6, 9, 11, 13, 14, 17, and 20.

The gene functions in clusters are the following:

**Cluster 1:**  Heat-shock proteins (>20), genes with catalytic activities (7), regulators of the cellular metabolic process (5), lipoxygenases (3), cysteine and methionine metabolism (3), ethylene signaling pathway (2);
**Cluster 2:**  Genes of ribosomal biogenesis (25), circadian rhythm regulators (3), sigma factors (3), FE superoxide dismutase 1 (*FSD1*), ANK6, which promotes the anthocyanin accumulator;
**Cluster 3:**  Genes responding to light stimulus (7), PIF TFs (4), phosphoproteins (4), phytochromes (3), cryptochromes (2), cryptochrome-interacting basic-helix-loop-helix (2), WRKY (2);
**Cluster 4:**  Genes of flavonoid biosynthesis (8), genes of phenylalanine biosynthesis (4), genes of the phenylpropanoid synthesis pathway (2), cytochrome P450 (*CYP98A3*);
**Cluster 5:**  Genes of arginine biosynthesis (3), asparagine synthetases (2), aspartate kinase;
**Cluster 6:**  Subunits of the LHCB complex (7), proteins of chlorophyll A-B binding family (4), subunits of photosystem (2);
**Cluster 7:**  Calcium-dependent protein kinases (3), respiratory burst oxidase (RBOHD);
**Cluster 8:**  Genes of glutathione transferases (9), dehydroascorbate reductase 1 (*DHAR1*), ascorbate peroxidase 2 (*APX2*);
**Cluster 9:**  Genes of the jasmonic acid biosynthetic process (4), WRKY regulators (3), acyl-coenzyme A oxidase 4 (*ACX4*), *ZAT7*;
**Cluster 10:**  Genes with ubiquitin protein ligase activity (4);
**Cluster 11:**  Genes of circadian rhythm (5), E3 ubiquitin ligase component complex (2), ethylene response DNA binding factor 2 (*RAV2*);
**Cluster 12:**  Transcription factors (5);
**Cluster 13:**  MAPK signaling pathway (2), myb domain protein r1 (*MYBR1*), 40S ribosomal protein S6 kinase (*S6K2*), ethylene responsive element binding factor 6 (*ERF6*);
**Cluster 14:**  Regulators of the jasmonic acid mediated signaling pathway (3);
**Cluster 15:**  Genes related to cysteine and methionine metabolism (5);
**Cluster 16:**  Wall-associated receptor kinase 2 (*WAK2*), transcription factor MYB51;
**Cluster 17:**  Phototropins (*PHOT1*/*PHOT2*, nonphototropic hypocotyl 3 (*NPH3*), root phototropism protein 2 (*RPT2*));
**Cluster 18:**  C3HC4-RING finger E3 ubiquitin ligase (*AtAIRP4*), Derlin-2/3 (*DER1*), ubiquitin fusion degradation protein 1 (*UFD1*);
**Cluster 19:**  Calcium-binding EF-hand family protein (*AT2G46600*), ATP-binding cassete G36 (*ABCG36*), calmodulin-like 4 (*TCH3*);
**Cluster 20:**  Hypersensitive to red and blue 1 (*HRB1*), serine/threonine phosphatase 7 (*PP7*),;
**Cluster 21:**  AICARFT/IMPCHase bienzyme family protein (*AT2G35040*), inositol 3-phosphate synthase (*MIPS2*).

**Figure 5:**
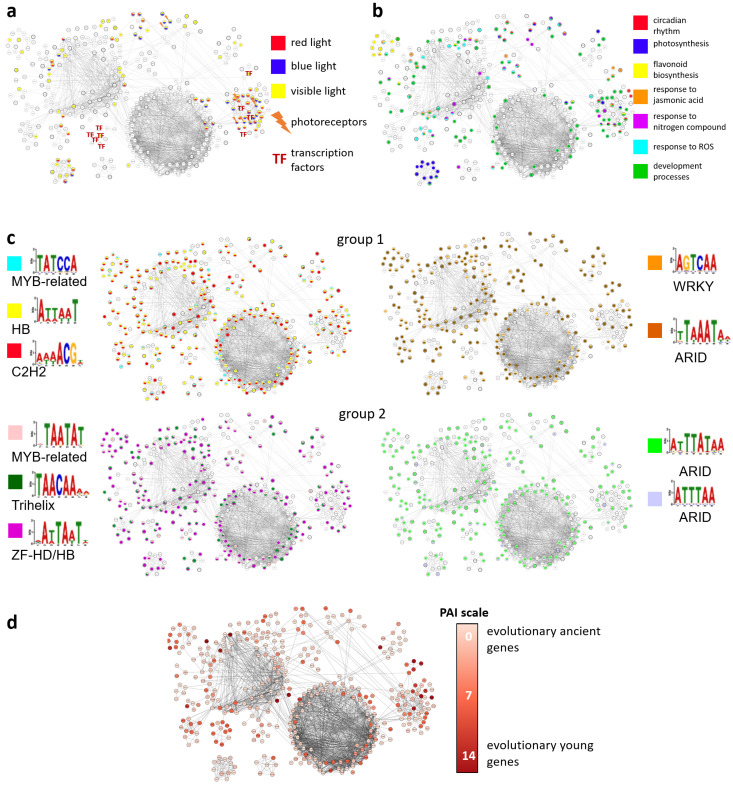
The high light response gene network layouts for distributions of (**a**) genes associated with red, blue, and visible light response, photoreceptors, and transcription factors; (**b**) genes associated with circadian rhythm, photosynthesis, flavonoid biosynthesis, response to jasmonic acid, nitrogen compound, and ROS, and developmental processes; (**c**) selected motifs; (**d**) phylostratigraphic index (PAI), low PAI corresponds to evolutionary ancient genes.

In addition, the distribution of light-related genes of the network is shown in Figure 5a, as well as selected metaterms’ distribution.

It should be noted that a significant decrease in the expression of cryptochromes (*CRY1*/*CRY2*) was detected, whereas the expression of phytochromes and phototropins did not reveal an unambiguous trend of change.

GO-terms involved in the response to high light stress, as well as their relations to clusters are shown in Figure 5a,b.

#### 2.3.2. Distribution of TFs Motifs over the Network

The distribution of identified TFs motifs is shown in Figure 5c. It is worth mentioning that homeobox and ARID families are the most distributed ones across the gene network. In addition, most of the motifs are distributed uniformly across all clusters (Figure 5c), which additionally confirms the high level of coordination in their regulation inside the reconstructed gene network.

#### 2.3.3. Distribution of PAI over the Network

The distribution of PAI across networks shows its overall homogeneous mode (Figure 5d), which testifies in favor of the similar evolution times of the main components of the network and the uniform functional evolution of most network modules. Clusters 1, 2, 5, and 6 are relatively ancient. Clusters 3, 4, 7, and 8 are relatively young.

## 3. Discussion

**Figure 6:**
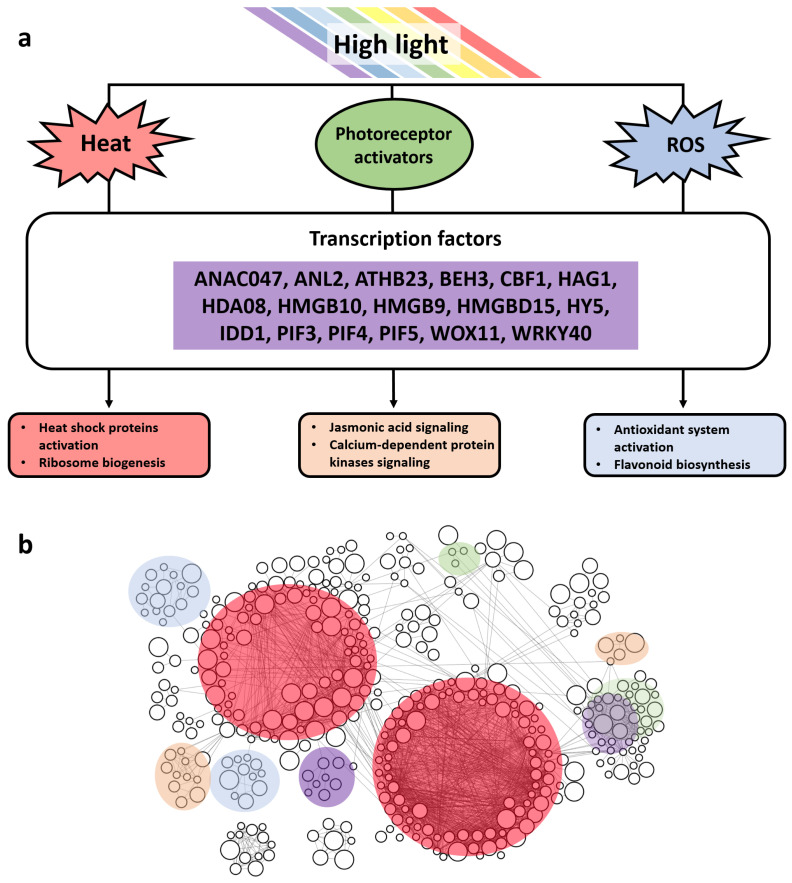
General scheme of molecular genetic mechanisms involved in high light response according to our meta-analysis. (**a**) Major high light response regulators; (**b**) the functional modules of the gene network identified as a result of the meta-analysis correspond to the main response pathways according to the color coding in (**a**).

The approach to the data meta-analysis in this study lies in the mainstream of methods that work with transcriptomic data and use a multilevel systematic approach. For example, the study [46] carried out a meta-analysis of microarray data in response to cold and drought that revealed general and stress-specific DEGs, as well as common GO-terms ‘photosynthesis’, ‘respiratory burst’, ‘response to hormone’, ‘signal transduction’, ‘metabolic process’, ‘response to water deprivation’; also, WRKY, NAC, MYB, AP2/ERF, and bZIP were identified as the main TF families involved in the stress response. Our analysis also identifies most of these families as major components of TFs involved in the high light response (Figure 6a). In addition, for the detection of shade-avoidance genes, authors performed a meta-analysis of 11 microarrays using ANOVA regarding various light conditions [47]; the authors revealed context-specific genes, among which, *HY5*, *PIF3*, *PIF4*, *PIF5*, *ARF6*, and *BZR1* were identified in the core component as key TFs. In our work, *HY5* and *PIFs* are also presented in the reconstructed network and in a selected dataset of DEGs; *HY5* is detected as upregulated, whereas *PIF4* is downregulated. In revealing the motives, the forerunner of our work is a meta-analysis of our colleagues, dedicated to the identification of regulatory motifs in response to various auxin concentrations and exposure times in *Arabidopsis thaliana* L. [48].

There are several works addressed that study the role of individual TFs in response to hyperinsolation (*ANAC032*, *MYB112*) [49,50]. Our meta-analysis of transcriptomic experiments reveals a specific component of the stress response associated mainly with cluster 3 in the light stress response gene network (Figure 4 and Figure 6b), in which, phytochromes and cryptochromes, as well as transcription factors *ATHB23* from the ZF-HD family (cluster 3) and *AT1G76110* from the ARID family (cluster 12), were detected (Figure 3b and Figure 5c). These transcription factors were confirmed as components of the reconstructed gene network and their targets were predicted across the network. The transcription factor *ATHB23* was shown as a component of the phytochrome-B-mediated red light signaling pathway [51]. In addition, this factor is involved in the gene regulatory network of roots development [52], so this factor could be a potential bridge between a high-light-mediated response and developmental adaptation processes.

In addition, we revealed that jasmonic acid is involved in regulating the high light response, and protein JAZ1 is a key transcription factor in this pathway [53]. Since the JAZ-mediated pathway is shown in multiple growth and developmental processes [54], this regulation could show the inter-functionality of this pathway in the case of a high light response. In addition, several works on *Arabidopsis thaliana* L. confirm the role of candidate transcription factors in relation to the light stress identified in our analysis. In particular, *MYB112* was shown to promote anthocyanin formation during high light stress [49]; bZIP transcription factor *HY5* was shown to be involved in response to light and ultraviolet-B radiation [55]; NAC transcription factor *ANAC078* was shown to regulate flavonoid biosynthesis under high light exposure [56,57]. In addition, we found that glutathione transferases often increase their expression in response to hyperinsolation (five out of six GST genes in the network) according to our analysis, which is in favor of the functional importance of the glutathione system in response to hyperinsolation, which is confirmed in a recent study of [58] on glutathione peroxidase 7.

Thereby, our analysis fits into the concept, confirming several existing works devoted to the identification of potential regulators in response to an increased illumination in *Arabidopsis thaliana* L. [57] at the systemic biological level, and allows for a comprehensive assessment of its molecular genetic regulation. In the future, setting up experiments on hyperinsolation and obtaining omics data for different plant species will make it possible to identify species-specific and interspecies’ molecular genetic subsystems of regulation. Another important fundamental issue is to identify the interplay between the response to hyperinsolation and growth/developmental processes.

## 4. Materials and Methods

### 4.1. Transcriptomic Data Search and Pre-Processing

The publicly available transcriptomic data were found in the NCBI GEO DataSets database (USA, Bethesda, Maryland, www.ncbi.nlm.nih.gov, date of access 5 January 2022) through a search with the following parameters:
Organism:Arabidopsis thaliana [porgn:__txid3702]Study type:Expression profiling by high throughput sequencingText fiters:“high light” OR “hyperinsolation”

The search resulted in 19 records being found. Among them, we manually verified those that were suitable for further meta-analysis using the following criteria: wild type *Arabidopsis thaliana* ecotypes (Columbia (Col-0), Landsberg erecta (Laer-0), or Wassilewskija (Ws-0)) were used in the experiment, high light of over 500 μmol m${}^{-2}$ s${}^{-1}$ acted as a stress factor in the treatment, samples selected for transcriptomic analysis contained aboveground parts of the plant. The search yielded 5 entries, as detailed in Table 1. The raw reads were normalized to CPM (counts per million) by dividing by the total size of sample libraries and multiplying by $1\times 10^{6}$.

### 4.2. Prediction of Differentially Expressed Genes in Individual Datasets

There are several techniques for conducting meta-analysis of transcriptomic experiments and assigning weights to differentially expressed genes: Fisher’s method, which sums up probability of logarithmic *p*-values from individual experiments [59]; maxP method, which takes maximum *p*-value for each DEG in analysis [60], and combined methods taking fold-change into consideration [61].

In the first step, we performed separate statistic analysis for each treatment using function *t*-test between control and stress replica. Then, we sorted genes from lowest to highest *p*-value and used step-down FDR-correction by multiplying *p*-value of the gene by step of comparison (FDR < 0.05). After that, we excluded genes that differed by less than 50% of average expression. As a result, we identified 5487 differentially expressed genes (DEGs), including groups of genes that increase and decrease expression. The number of DEGs for each GSE ID is stated in the Table 1. Statistic analysis was peformed using R language (Vienna, Austria) built-in functions.

For combining DEGs from multiple transcriptomic studies, we used the following meta-analysis methods:

1. Preferential selection of genes by the level of their changes in single experiments:
  (1) $$w_{i}=\sum_{i=1}\left|log_{2}\Delta_{expr}\right|\cdot r,$$
  where
  (2) $$\begin{array}{ccc} r & =\; & \left\{\begin{array}{cc} 4 & \mathrm{if}\;FDR\leq 0.01,\\ 2 & \mathrm{if}\;0.01<FDR\leq 0.05,\\ 1 & \mathrm{if}\;0.05<FDR\leq 0.25,\\ 0 & \mathrm{otherwise}. \end{array}\right. \end{array}$$
2. Preferential selection of genes by their presence in different experiments
  (3) $$w_{2}=n\cdot \sum_{i=1}\left|log_{2}\Delta_{expr}\right|\cdot r$$
3. Preferential selection of genes by combined Fisher’s *p*-value above all detected experiments and their summary change in their detection in experiments
  (4) $$w_{3}=|log_{10}p-value|\sum |log_{2}\Delta_{expr}|$$

The intersection of these three methods revealed a set of 1151 selected DEGs (see Supplementary Table S3) that were used in further analysis.

### 4.3. Prediction of TFs Families Enriched in Individual Datasets and Matching Them with Experimental Conditions

For determining the enrichment of TFs binding sites in a given set of gene promoters, we used the procedure described in [35]. The output of the procedure is the list of TF, which could potentially regulate input set of genes. 568 genome-wide DAP-Seq profiles for 387 *Arabidopsis thaliana* L. TFs, containing binding peaks of TFs, were downloaded from the Plant Cistrome Database (USA, San Diego, California) [37]. We used [–1500; +1] upstream regions of 19916 protein-coding genes (TAIR10 genome release, USA, Newark, New Jersey [62]) as the promoters’ background. Promoters of the input DEGs for each experimental point, considering the direction of expression change, were used as a foreground. For each TF profile, we calculated number of DEGs that contain binding peaks in their promoters. To calculate the enrichment of mapped peaks in the foreground promoters, we compared them to the background one. We assessed the significance of TF potential regulation by Fisher’s exact test with the correction of a multiple correction by FDR threshold of 0.05 (Benjamini–Yekutelli method [63]).

The conditions of the experimental points were systematized into a matrix containing numerical characteristics on the intensity, duration of light exposure, ecotype of samples, tissue, plant age, and direction of change in genes expression. The TF matrix for each experimental point includes the frequencies of occurrence of TF families separately for groups of genes that increase and decrease expression. For these two matrices (see Supplementary Table S4), two-block partial least squares (2B-PLS) analysis was used in accordance with the algorithm [64] implemented in the program PAST 3.0 (PAlaeontologica STatistics, ver. 1.74, Norway, Oslo [65]). Plotting PLS scores were also carried out using PAST 3.0.

### 4.4. Identification of Enriched Motifs in Promoters of Selected DEGs

For revealing specific motifs associated with selected DEGs in first stage, we used approach originally described in [34] and the package metaRE (Russia, Novosibirsk, https://github.com/cheburechko/metaRE, date of access 5 January 2022 [66]). Analysis algorithm included the following steps. (i) Creating two binary matrices (upregulated / downregulated) of DEGs, where columns are experiments and rows are genes. Matrix cells contain information about the state of gene in every experiment (DEG or non-DEG). (ii) Uploading to metaRE a set of 27628 1500bp-promoter regions for all *Arabidopsis thaliana* L. genes obtained from TAIR database (https://www.arabidopsis.org/portals/genAnnotation/genome_annotation_tools/cis_element.jsp, date of access 5 January 2022). (iii) Calculation of meta *p*-value adjusted with FDR criterion *p*-value for presence of each of 2080 variants of hexamers in the set of selected DEGs. (iv) Selection of hexamers with FDR *p*-value < 0.05 and permutation *p*-value < 0.05. As a result, we obtained a set of 61 hexamers (see Supplementary Table S5).

Promoters 1500 bp upstream of TSS from 1151 selected DEGs were scanned for presence of 61 hexamers (*p*-value < 0.05). Occurrences of 61 hexamers were counted in a 50 bp sliding window with 5 bp increment using stringr and Biostrings R packages. Fragments of varied length with hexamer count > 1 were extracted and scanned for ungapped motifs that are relatively enriched compared to the background set of all *Arabidopsis thaliana* L. promoters using STREME (MEME suite 5.4.1, Australia, Queensland [38]). The found promoter regions and their coordinates are indicated in the Supplementary Tables S6 and S7. Enriched motifs passing the significance threshold (e-value < 0.005) were compared to ArabidopsisDAPv1 [37] database using TOMTOM tool (Australia, Queensland [39]) containing DAP-seq [37] and PBM (Spain, Madrid [67]) databases for *Arabidopsis thaliana* L. As a result, we obtained a set of 10 selected motifs (see Supplementary file S10).

For clustering analysis of selected motifs, we used the function cluster.hierarchy.ward from SciPy library with distance matrix constructed as binary matrix, where 1 means presence of a particular motif in the promoter of a specific gene.

### 4.5. Gene Ontology Enrichment Analysis

For the set of selected DEGs, the enrichment of GeneOntology terms (GO-terms), including their network, was revealed using ShinyGO service v. 0.75 (https://bioinformatics.sdstate.edu/go, date of access 5 January 2022 [41]). Network was reconstructed with parameter edge cutoff = 0.5. We have identified 174 terms, which are grouped into 33 clusters (see Supplementary Table S9).

### 4.6. Reconstruction of the Gene Network

For reconstruction of the gene networks, we used a set of genes with known association with the light stress (151 genes) referred from the article of [31]. This reference set was combined with selected DEGs revealed from transcriptomic meta-analysis (1151 gene). For the resultant set in the STRING database (https://string-db.org/, date of access 5 January 2022 [40]), we found 1860 protein–protein interactions. The following parameters of STRING search were used:

Sources of interaction: confirmed experimentally and found in databases;

Threshold of combined interaction score: medium (0.4).

Obtained gene network was exported to Cytoscape environment v. 3.7.2 [68] for further layout (GO and TFs), visualization, and exporting the figures. For the final version of the gene network, we exclude nodes with no connections and small elements that have less than 3 internal connections. As a result, we obtained the gene network with 382 verticies and 1812 edges (input table for Cytoscape is presented in Supplementary Table S8).

To analyze the network topology, we used NetworkAnalyzer (https://apps.cytoscape.org/apps/networkanalyzer, date of access 5 January 2022 [69]). For initial layout, we used the parameter EdgeBetweenes and built-in algorithm Prefuse Force Directed OpenCL Layout. Thus, three largest clusters of network were identified. After, we used ClusterOne algorithm to identify minor clusters with minimal size = 3 (https://paccanarolab.org/static_content/clusterone/cl1-cytoscape-1.0.html, date of access 5 January 2022 [70]) and made the final layout manually.

To estimate the evolutionary age of nodes in the gene network, the phylostratigraphic age index (PAI) metric was used (reference work by [31], identity parameter = 0.6). This metric makes it possible to estimate the evolutionary age of a node: low PAI values (PAI < 7) correspond to evolutionarily ancient nodes.

## 5. Conclusions

In this work, we present the results of a large-scale systematic analysis of the high light response regulation at different levels in *Arabidopsis thaliana* L. We aimed to consider all available aspects of regulation for the molecular genetic system under study; therefore, we integrate data on changes in the transcriptomic profile, protein–protein interactions, and regulatory motifs that reflect the action of transcription factors, also involving data on the functional annotation of genes. This integration used a variety of methods operating on multidimensional data. A meta-analysis of transcriptomic experiments revealed a set of 1151 differentially expressed genes (DEGs) that most stably and significantly change behavior in response to stress. Based on this set of genes, we reconstructed the resulting core gene network with functional modules reflecting both the specific response, regulation of the non-specific response, and the global metabolic change in response to high light stress. The combined structural analysis of the functional modules in this gene network and the massive analysis of data on the structure of promoter regions predicted the composition of major transcription factors and the features of their resulting action associated with the direction of DEGs’ change in expression, as well as the intensity and duration of high light exposure. However, these predicted components of the regulatory system in *Arabidopsis thaliana* L. still require versatile experimental verification. Nevertheless, the reconstructed gene network, the structure of its functional modules, and candidate transcription factors serve as a significant basis for understanding the functioning and evolution of the genetic response system to high light in plants.

## Figures and Tables

**Table 1:** Transcriptomic datasets related to different high light treatments of *Arabidopsis thaliana* L. used for the current meta-analysis.

GEO ID	Ecotype	Sampled Tissue	Plant Age	PPFD, μmol m${}^{-2}$ s${}^{-1}$	Treatment Duration	Control/ Treatment Samples	DEGs ^1^	Related Article
GSE111062	Col-0	seedling	7 days	1200	0.5–72 h	12/12	595	[42]
GSE117296	Col-0	leaf	4–5 weeks	2000	2, 4, 8 min	6/18	2654	[43]
GSE117298	Col-0	leaf	4–5 weeks	2000	8 min	6/6	633	[43]
GSE134391	Col-0	leaf	30 days	600	7 h	3/3	492	[44]
GSE132626	Col-0, Ws-0	leaf	NA ^2^	500	15–240 h	3/15	1113	[45]

^1^ FDR < 0.05, ^2^ NA—not avaliable.

**Table 2:** Characterization of Clusters in the Light Stress Response Gene Network.

ID	Vertices	Edges	↑ ^1^	↓ ^2^	↑↓ ^3^	Added
Cluster 1	108	420	43	16	38	11
Cluster 2	97	983	15	13	67	5
Cluster 3	48	87	11	15	6	16
Cluster 4	15	22	3	7	4	0
Cluster 5	14	20	6	5	3	0
Cluster 6	13	59	2	4	3	4
Cluster 7	11	30	7	3	0	1
Cluster 8	11	14	5	1	3	2
Cluster 9	9	8	7	0	2	0
Cluster 10	7	21	6	1	0	0
Cluster 11	7	7	0	2	2	3
Cluster 12	7	7	3	2	0	2
Cluster 13	6	6	6	0	0	0
Cluster 14	5	6	5	0	0	0
Cluster 15	5	5	1	3	1	0
Cluster 16	5	4	1	1	2	0
Cluster 17	4	3	0	2	0	2
Cluster 18	3	2	2	0	1	0
Cluster 19	3	2	3	0	0	0
Cluster 20	2	1	0	0	0	2
Cluster 21	2	1	0	0	2	0

^1^ upregulating genes, ^2^ downregulating genes, ^3^ multidirectional change in expression.

## Data Availability

Not applicable.

## Supplementary Materials

The following are available online at https://www.mdpi.com/article/10.3390/ijms23084455/s1.

## Abbreviations

The following abbreviations are used in this manuscript:

2B-PLS	Two-block partial least squares
ABA	Abscisic acid
AP2	APETALA2
ARID	AT-rich interaction domain
bZIP	Basic leucine zipper domain
BZR	Brassinazole-resistant family
C2H2	Cys2His2
CPM	Counts per million
DAP-seq	DNA affinity purification sequencing
DEG	Differentially expressed gene
ELIP	Early light-induced protein
EREBP	Ethylene-responsive element binding protein
FDR	False discovery rate
GEO NCBI	Gene Expression Omnibus National Center for Biotechnology Information
GO	Gene ontology
HB	Homeobox family
HSF	Heat stress transcription factor family
JA	Jasmonic acid
PAI	Phylostratigraphic age index
PIF	Phytochrome interacting factor
PPFD	Photosynthetic photon flux density
ROS	Reactive oxygen species
TF	Transcription factor
UV-B	Ultraviolet B
UVR8	UV-B resistance 8
ZF-HD	Zinc finger homeobox family protein
