# Supplementary material for: Transcriptomic Data Meta-Analysis Sheds Light on High Light Response in Arabidopsis thaliana L
Source: Int J Mol Sci. 2022 Apr 18;23(8):4455. doi: 10.3390/ijms23084455 (PMC9026532; doi:10.3390/ijms23084455)
Supplement: Supplementary file 1 [file ijms-23-04455-s001.zip › S1.pdf]

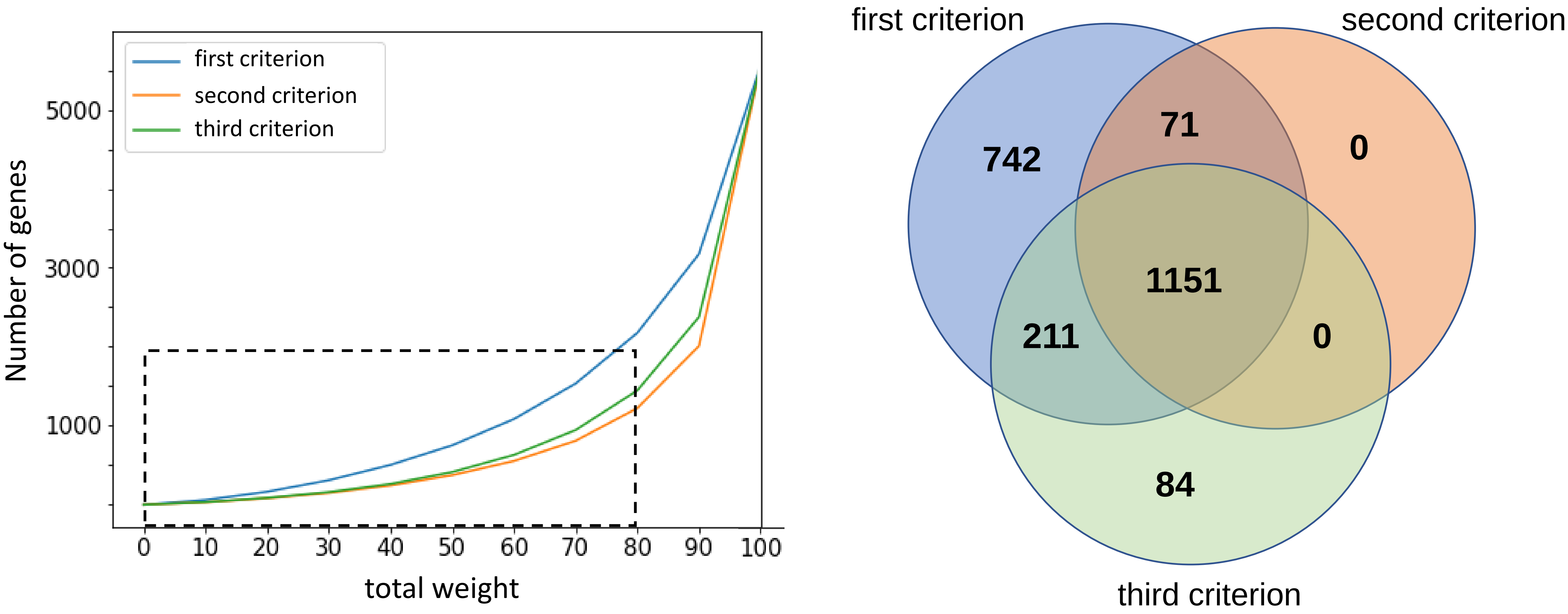

Figure S1. Results of ranking of differentially expressed genes in hyperinsolation by three criterion used.
On the left part is graph of distribution of weights among all DEG's of total weights. The most informative area (which contains 80% of total weight by each used criterion) which was used for final selection is highlighted by dotted line. On the right part if Venn diagram which contains intersection genes between three criteria. Finally selected genes for further analysis contained in a central intersection (1151).
