# Supplementary material for: Transcriptomic Data Meta-Analysis Sheds Light on High Light Response in Arabidopsis thaliana L
Source: Int J Mol Sci. 2022 Apr 18;23(8):4455. doi: 10.3390/ijms23084455 (PMC9026532; doi:10.3390/ijms23084455)
Supplement: Supplementary file 1 [file ijms-23-04455-s001.zip › S10.pdf]

STREME
Sensitive, Thorough, Rapid, Enriched Motif Elicitation

For further information on how to interpret these results please access https://meme-suite.org/meme/doc/streme.html.
To get a copy of the MEME software please access https://meme-suite.org.

If you use STREME in your research, please cite the following paper:
Timothy L. Bailey, "STREME: accurate and versatile sequence motif discovery", *Bioinformatics*, Mar. 24, 2021. [full text]

Discovered Motifs | Inputs & Settings | Program Information | Motifs in MEME Text Format | Matching Sequences NEW | Results in XML Format

Discovered Motifs

Next Top

Motif	Logo	RC Logo	P-value	E-value NEW	Sites	More	Submit/Download	Positional Distribution NEW	Matches per Sequence NEW
1-WATTAATW	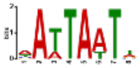	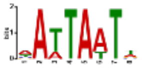	8.9e-023	2.0e-021	1746 (35.4%)	↧	⇢	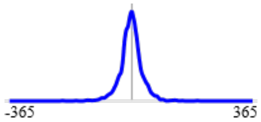	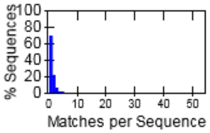
2-ATTTATAA	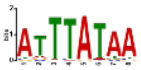	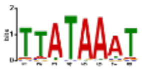	1.0e-014	2.4e-013	1483 (30.0%)	↧	⇢	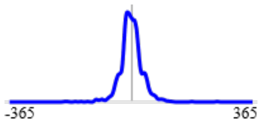	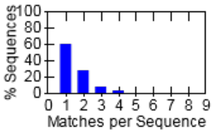
3-TTAAATAW	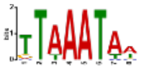	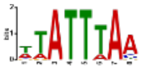	1.4e-009	3.1e-008	1387 (28.1%)	↧	⇢	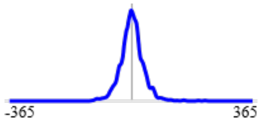	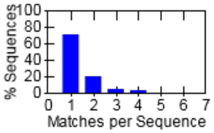
4-MTAATAT	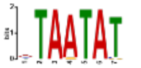	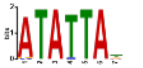	2.8e-008	6.3e-007	674 (13.6%)	↧	⇢	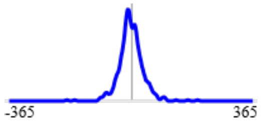	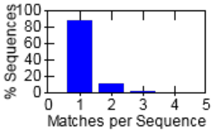
5-ATTAAT	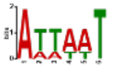	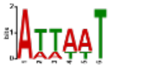	5.9e-008	1.4e-006	1660 (33.6%)	↧	⇢	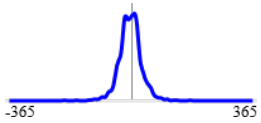	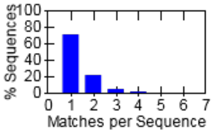
6-TATCCA	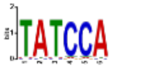	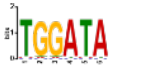	4.7e-005	1.1e-003	353 (7.1%)	↧	⇢	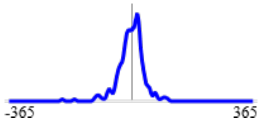	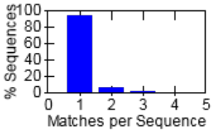

Stopped because 3 consecutive motifs exceeded the p-value threshold (0.05).
STREME ran for 1667.29 seconds.

Motif	Logo	RC Logo	P-value	E-value	Sites	More	Submit/Download	Positional Distribution	Matches per Sequence
7-ATTTAA	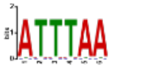	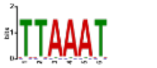	9.9e-005	2.3e-003	211 (4.3%)			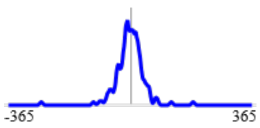	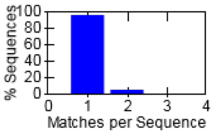
8-AWAACGW	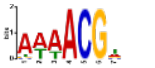	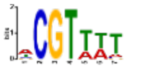	3.0e-004	7.0e-003	1021 (20.7%)			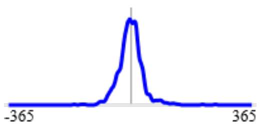	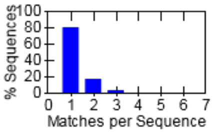
9-AGTCAA	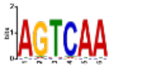	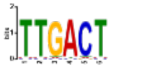	8.7e-004	2.0e-002	466 (9.4%)			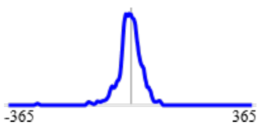	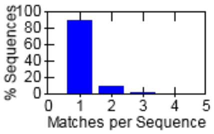
10-TAACAAAA	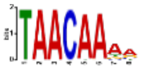	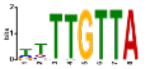	1.4e-003	3.3e-002	568 (11.5%)			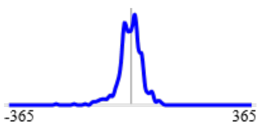	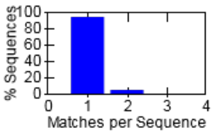
11-MCACGTDAY	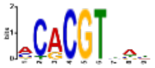	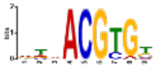	4.1e-003	9.5e-002	716 (14.5%)			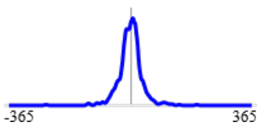	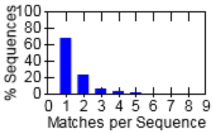
12-CATTTAV	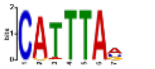	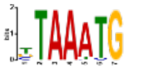	9.7e-003	2.2e-001	406 (8.2%)			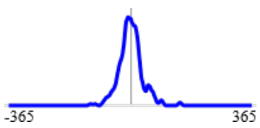	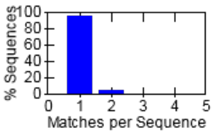
13-ATTATAAT	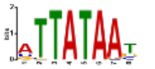	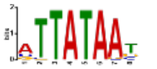	1.2e-002	2.8e-001	87 (1.8%)			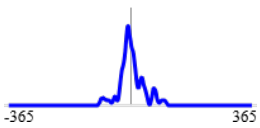	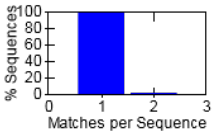
14-TCTAGA	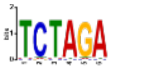	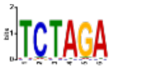	1.3e-002	3.1e-001	31 (0.6%)			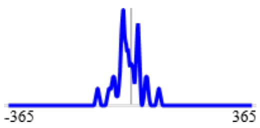	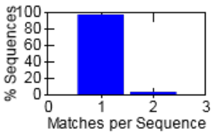

Stopped because 3 consecutive motifs exceeded the p-value threshold (0.05).
STREME ran for 1667.29 seconds.

Motif	Logo	RC Logo	P-value	E-value NEW	Sites	More	Submit/Download	Positional Distribution NEW	Matches per Sequence NEW
15-ATATAC	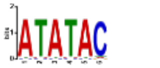	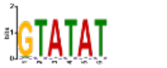	1.5e-002	3.4e-001	584 (11.8%)			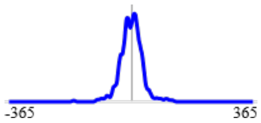	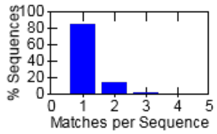
16-TATTAAAAA	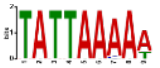	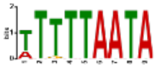	2.3e-002	5.3e-001	52 (1.1%)			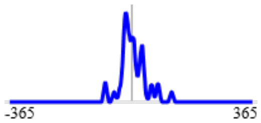	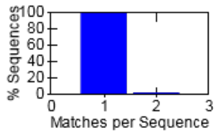
17-NHTCTAGADN	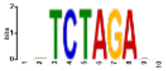	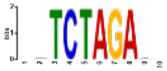	2.6e-002	5.9e-001	257 (5.2%)			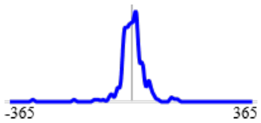	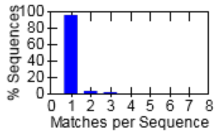
18-YACGWAW	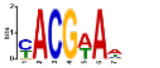	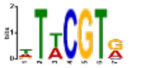	3.0e-002	6.9e-001	356 (7.2%)			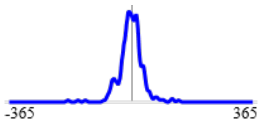	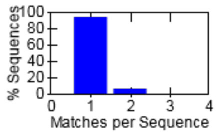
19-RRACCATTA	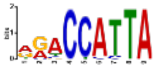	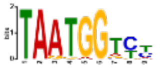	4.6e-002	1.1e+000	99 (2.0%)			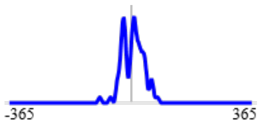	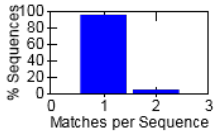
20-AGTCTTHW	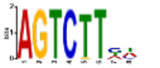	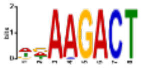	4.9e-002	1.1e+000	275 (5.6%)			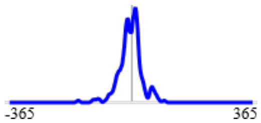	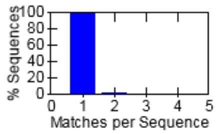
21-AAARACTTTRTTKTAAA	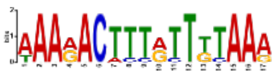	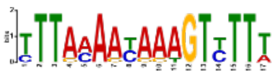	1.3e-001	3.0e+000	9 (0.2%)			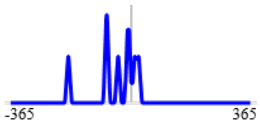	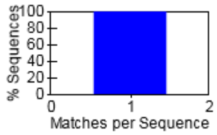
22-GTGACRWA	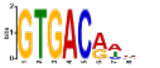	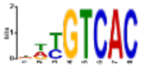	2.4e-001	5.4e+000	253 (5.1%)			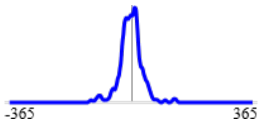	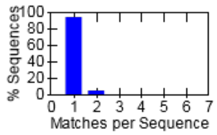

Stopped because 3 consecutive motifs exceeded the p-value threshold (0.05).
STREME ran for 1667.29 seconds.

Motif	Logo	RC Logo	P-value	E-value	Sites	More	Submit/Download	Positional Distribution	Matches per Sequence
23-ACAAGT	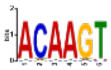	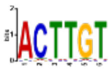	2.8e-001	6.5e+000	422 (8.5%)	↧	⇢	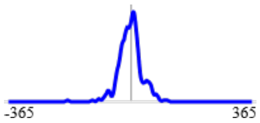	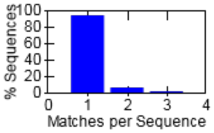

Stopped because 3 consecutive motifs exceeded the p-value threshold (0.05).
STREME ran for 1667.29 seconds.

INPUTS & SETTINGS

Previous Next Top

Sequences

Role	Source	Alphabet	Sequence Count	Total Size
Positive (primary) Sequences	regions-7.fasta	DNA	4939	480645
Negative (control) Sequences	prom-1500.fasta	DNA	32805	3198248

Background Model

Source: built from the negative (control) sequences

Order: 2 (only order-0 shown)

Name	Freq.	Bg.				Bg.	Freq.	Name
Adenine	0.351	0.33	A	~	T	0.33	0.351	Thymine
Cytosine	0.149	0.17	C	~	G	0.17	0.149	Guanine

Other Settings

**Strand Handling**	Both the given and reverse complement strands are processed.
**Objective Function**	Differential Enrichment
**Statistical Test**	Binomial Test
**Minimum Motif Width**	6
**Maximum Motif Width**	20
**Test Set**	10% of the input sequences were randomly assigned to the test set.
**Word Evaluation**	Up to 25 words of each width from 6 to 20 were evaluated to find seeds.
**Seed Refinement**	Up to 4 seeds of each width from 6 to 20 were further refined.
**Refinement Iterations**	Up to 20 iterations were allowed when refining a seed.
**Random Number Seed**	1
**Total Length**	The total length of the sequence set(s) was not limited.
**Maximum Motif p-value**	Stop when the p-value is greater than 0.05 for 3 consecutive motifs.
**Maximum Motifs to Find**	No maximum number of motifs.
**Maximum Run Time**	No maximum running time.

Previous Top

STREME version
5.4.1 (Release date: Sat Aug 21 19:23:23 2021 -0700)

Reference
Timothy L. Bailey, "STREME: accurate and versatile sequence motif discovery", *Bioinformatics*, Mar. 24, 2021. [full text]

Command line

```
streme --p regions-7.fasta --n prom-1500.fasta --o ./streme_out-7_005-1148 --dna --minw 6 --maxw 20 --seed 1
```
